# Single punch, double biopsy

**DOI:** 10.1186/s40064-016-3141-1

**Published:** 2016-08-30

**Authors:** Malte Krönig, Norbert Nanko, Vanessa Drendel, Martin Werner, Wolfgang Schultze-Seemann, Anca L. Grosu, A. Cordula Jilg

**Affiliations:** 1 Department of Urology, University of Freiburg Medical Centre, Hugstetter Strasse 55, 79106 Freiburg, Germany; 2 Department of Radiation Oncology, University of Freiburg Medical Centre, Hugstetter Strasse 55, 79106 Freiburg, Germany; 3 Department of Clinical Pathology, University of Freiburg Medical Centre, Breisacher Strasse 155a, 79106 Freiburg, Germany

**Keywords:** Needle biopsy, Prostate cancer, Primary tissue, Tissue heterogeneity, Single cell

## Abstract

**Objective:**

In lethal primary metastatic prostate cancer, biopsy material is often the only accessible cancer tissue. Lack of tissue quantity limited the use of biopsy cores for analyzing higher numbers of molecular markers and standard histopathologic evaluation for clinical diagnosis simultaneously. Recent advances in single cell analytics have paved the way to characterize a tumor in more depth from minute input material such as biopsies. We therefore aimed to develop a biopsy needle, which generates two cores side by side from the same punch: one for standard histopathologic analysis to allow for routine diagnostics and the second one for single cell analytics.

**Methods:**

On the basis of a conventional punch biopsy needle we have milled two parallel longitudinal rifts into the needles shat which are separated by a 100 µm thick metal sheet. Each rift can harbor a single tissue core.

**Results:**

Two cores from the same punch were generated reproducibly from a radical prostatectomy specimen and showed congruent results in histopathologic analysis. Both cores yielded equally sufficient material for standard H&E staining and histopathological evaluation.

**Conclusion:**

Our modified biopsy system will allow for simultaneous acquisition of tissue cores for diagnostic and scientific analysis from solid tumors or metastatic sites.

## Background

There still exists a discrepancy between the clinical diagnosis or treatment and growing molecular knowledge of prostate cancer. Gold standard for the diagnosis of prostate cancer is the histopathologic analysis of prostate needle biopsies. They often represent the only available tumor tissue because non-surgical systemic treatment such as radiation or androgen deprivation therapy (ADT) is administered to these patients. Only few parameters are analysed such as morphology (Gleason Score), tumour volume (number of tumour bearing biopsy cores and percentage of tumor tissue in all positive biopsy cores) (Schroder et al. 2012) and in some cases protein expression (AMACR and p63) (D’Amico et al. 1998; Heidenreich et al. 2014; b; Jiang et al. 2002; Rubin et al. 2002; Zhou et al. 2002). In localized prostate cancer risk stratification systems are also based on these few parameters and lead to significant inaccuracies in clinical endpoints (Schroder et al. 2012). In primary metastatic disease resistance to systemic ADT still hampers long lasting therapeutic success. Risk stratification systems based on the same diagnostic parameters do not allow for predictions regarding time to resistance or response to specific ADT regimes. Despite systemic therapy survival at this stage is significantly compromised and novel therapeutic concepts are urgently needed. To overcome the diagnostic and prognostic variability as wells as the therapeutic limitations by current standards, molecular strategies have to be developed for improved diagnostic and therapeutic purposes. Crucial to such undertaking are adequate tissue samples from primary and metastatic sites. Tissue biopsy cores will remain the primary source of such tissue samples. The limited tissue quantity could be compensated by high flexibility and wide availability to biopsy nearly every tissue within the body with acceptable risk for the patient. Furthermore, recent advances in single cell analytics have paved the way to utilize even minute amounts of input material (Kronig et al. 2015; Lohr et al. 2014; Patel et al. 2014; Shalek et al. 2014; Trombetta et al. 2014). Prostate cancer is no longer seen as a standalone cell type but rather has to be handled as a complex network of various cell types influencing tumour initiation, progression and therapy (Lohr et al. 2014; Haffner et al. 2013; Brennen et al. 2012; Comito et al. 2014; Donkor et al. 2011; Karja et al. 2005; Madar et al. 2013; Maher and Davies 2004; Sfanos et al. 2008; Sluka and Davis 2013; Webber et al. 2015). To resolve the cellular heterogeneity and to identify intercellular networks single cell analytics represent a powerful tool (Lohr et al. 2014; Patel et al. 2014; Shalek et al. 2014; Trombetta et al. 2014; Pettit et al. 2014; Picelli et al. 2013; Ramskold et al. 2012). The limited tissue quantity generated by biopsies turns into an advantage and even potentiates by the ability for high sampling frequency. Primary biopsy cores contain viable cells, which provide optimal results with regard to downstream analytics. Gold standard histopathologic analysis is usually performed on formalin fixed tissue, which provides optimal conditions for morphologic analysis but limited capacity for large scale single cell analytics. Nonetheless, gold standard histopathologic analysis must not be compromised by using primary biopsy tissue within research project. We therefore aimed to develop a biopsy needle, which generates two parallel cores out of one single punch: one for standard histopathologic analysis to allow for routine diagnostics and the second one for single cell analytics.

## Methods

### Needle modification

We used a MaxCore System (MC1416) by BARD Biopsy Systems: penetration depth 22 mm, length and width of the original biopsy rift: 19 × 2.1 mm. In a first step the original needle notch including the rift was cut away which shortened the needle by ca. 22 mm. We then manufactured a positioning frame to keep the needle under tension and mounted it on a fine metal milling machine. Two parallel 19 mm in length rifts were then milled into the needle’s shaft in such a way that a 100 µm thin sheet remained standing between them. The tip of the needle was then sharpened in the original angle to reconstitute a fully functioning needle. The cutting cylinder, which is running over the needle was adapted to the new needle length and the notch sharpened accordingly. No other modification to the biopsy system were necessary. The modifications were neither initiated by BARD Biopsy Systems nor are they covered by the company’s liability regulations. The modified biopsy system is not certified for in patient use. Prototype testing has been performed with ex vivo radical prostatectomy samples within this research project approved by our local ethics committee (328/15). Samples were biopsied within 15 min after resection. The patient gave written consent prior to the procedure.

### Histologic analysis

Fixation, paraffin embedding, cutting, H&E staining and Gleason grading was performed according to routine diagnostic standards.

## Results

**Fig. 1:**
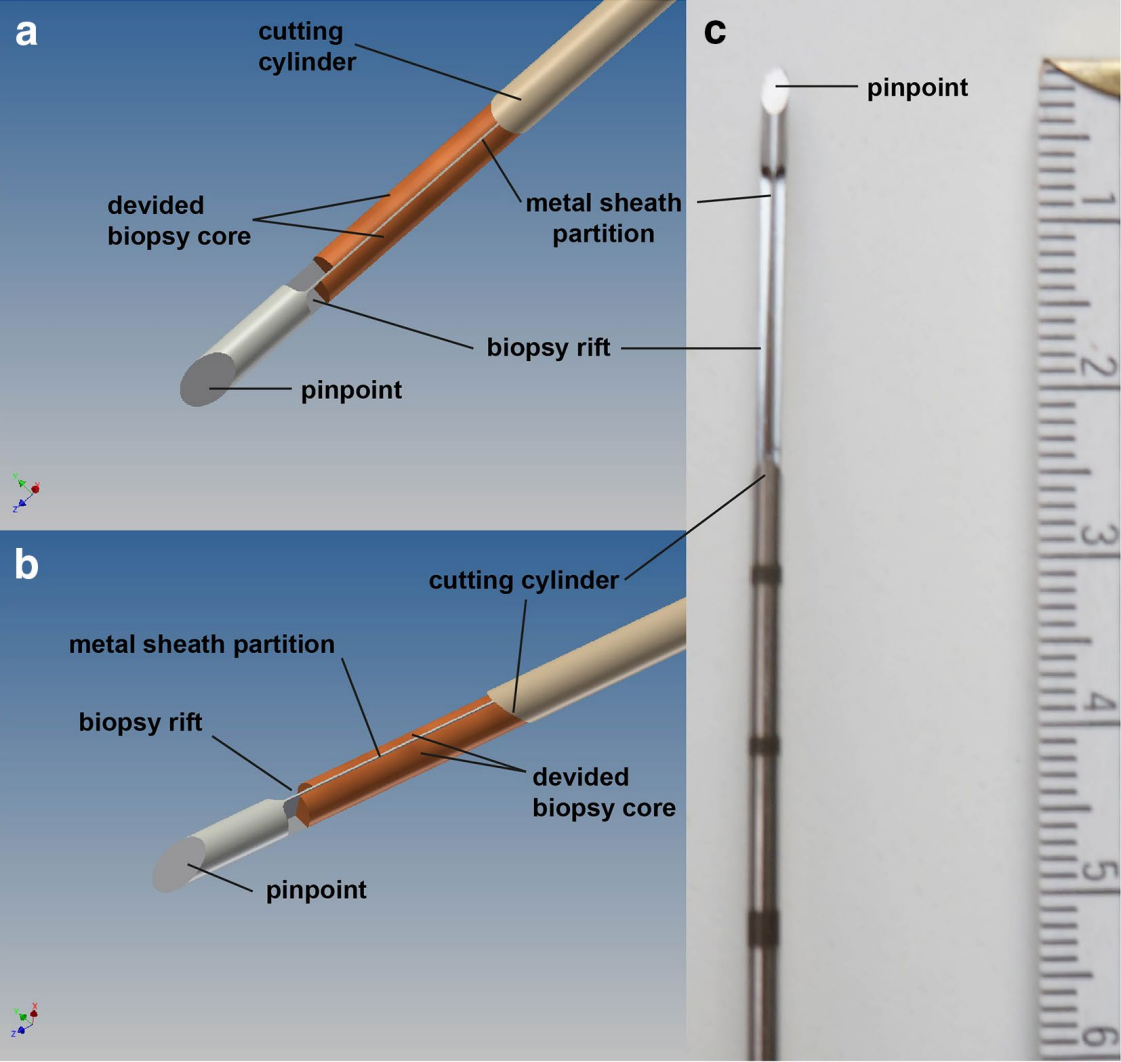
**a**, **b** Schematic illustration of the modified biopsy needle from above (**a**) and side angled (**b**); **c** photo from the prototype needle. A 100 µm thick longitudinal metal sheath was carved out to generate two parallel rifts to harbor the biopsy cores. No other modifications to the system was necessary to allow for normal function. *Scale* is indicated in cm

**Fig. 2:**
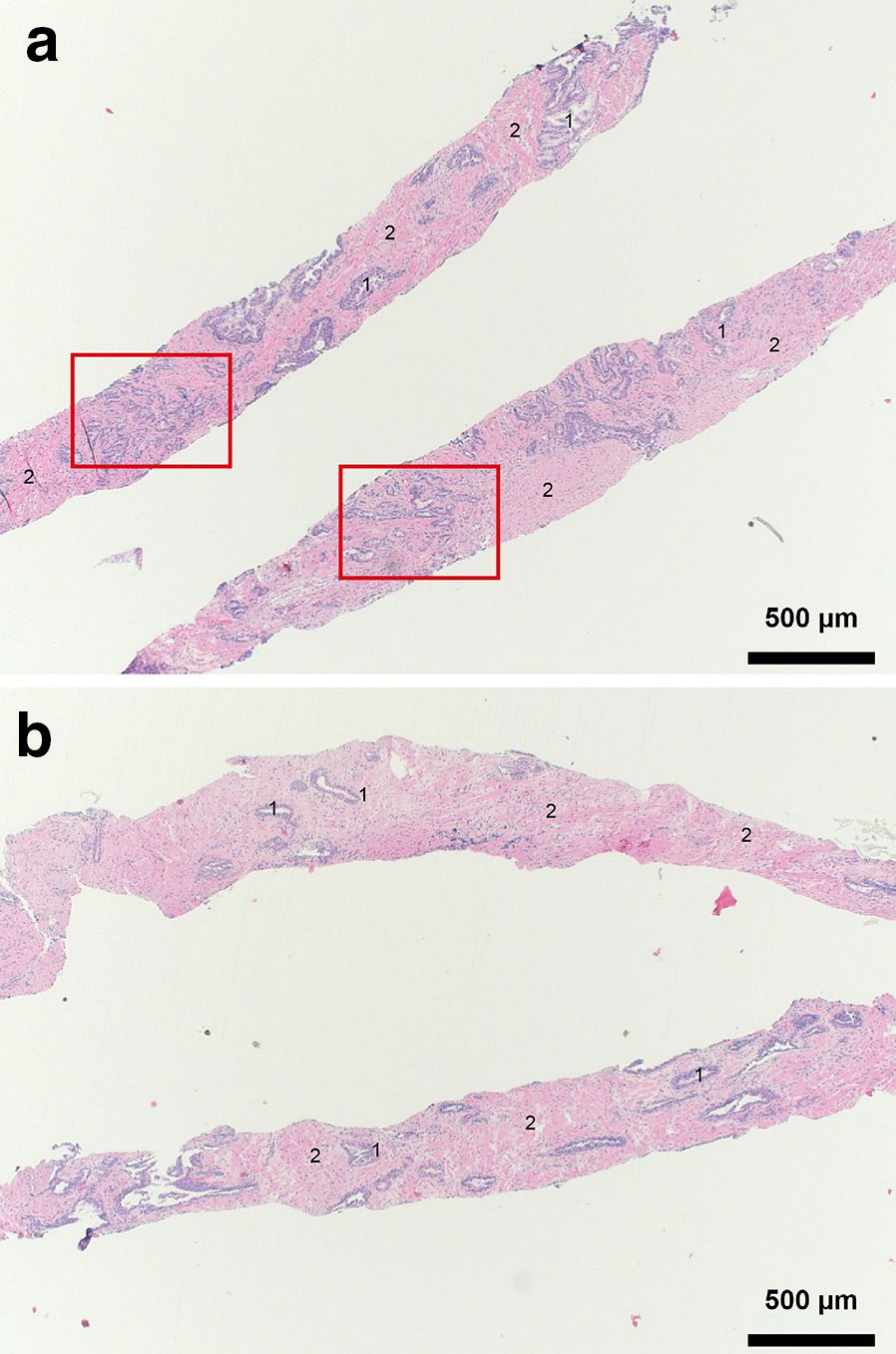
**a** H&E staining of the two parallel biopsy cores. *Red squares* show tumor cells in both cores, but also benign cells (*1*) and mesenchymal cells (*2*); **b** H&E staining of the two parallel biopsy cores. No tumor cells are present. Benign cells (*1*) and mesenchymal cells (*2*) are equally distributed in both cores; *scale bar* = 500 µm

We successfully modified the original needle by milling two parallel rifts separated by a 100 µm thin sheet into the needles shaft (Fig. 1a–c). The original single rift was cut way. The function of the entire biopsy system was not compromised. The core size was ca. 19 × 1 mm each versus 19 × 2.1 mm of the single core. Double biopsy cores were easily removed from the biopsy needles. The needle could also be reused for several biopsies from the same patient sample. The quality of the biopsy cores was sufficient to allow for standard H&E staining within routine diagnostic workup. All biopsy cores were generated from the same prostatectomy specimen: a 57 year old male patient with pre-operative PSA 7.65 ng/ml, Overall Gleason Score was 3 + 4 = 7. Ten double biopsies were taken in total. Cores were fixed, paraffin embedded, cut and routine H&E staining was performed. Cores were analyzed for tumor or no tumor. In cases of tumor Gleason Grading was assigned to each core. 6 of the 10 double biopsies were tumor bearing and 4 were not. All 10 double cores showed congruent results. Two double cores are shown here. Both cores in Fig. 2a show prostate cancer Gleason Score 3 + 4 (Grade Group 2) in ca. 10–20 % of the biopsy core, which corresponds to ca. 1.5–2.0 mm of tumor. Both cores in Fig. 2b show benign prostate glands (1). The mean core length was 15 mm (±3 mm). Special attention was payed during paraffin embedding to allow for optimal cutting. The cellular heterogeneity of prostate cancer and benign prostate tissue is also underlined these representative biopsy cores. Tumor cells in Fig. 2a (red square) represent the minority of cells (20 %, 2 mm of tumor). The tissue is dominated by mesenchymal cells (2) which comprise stromal cells, endothelial cells, myofibroblasts and lipocytes. But also benign prostate glands are located in close proximity to the tumor cells. Also the benign sample (Fig. 2b) is dominated by mesenchymal cells (2).

## Discussion

Our modified biopsy needle enables the simultaneous generation of two biopsy cores side by side from a single punch. The technique ensures histological evaluation of the tissue and at the same time provides a second core for molecular analysis down to the single cell level. No further modifications to the biopsy system were necessary. It is a universally applicable cost effective technique in several solid tumor entities and organs. To the best of our knowledge no comparable system is available on the market or has been described in the literature.

Alternative approaches which used formalin fixed paraffin embedded tissue (FFPE) are limited by the output yield of DNA, RNA or protein and do not allow for in depth single cell analytics (Hedegaard et al. 2014). Fresh tissue on the other allows for utilizing all molecular techniques scaled for low input material, even culturing of the cells is possible (Kronig et al. 2015; Lohr et al. 2014; Patel et al. 2014; Shalek et al. 2014; Picelli et al. 2013; Ramskold et al. 2012; Treutlein et al. 2014). We have previously shown that needle biopsy cores can utilized for single cell gene expression analysis and culturing (Kronig et al. 2015).

Even from the few representative biopsy cores shown here it is evident that bulk tissue analysis will produce significantly biased results due to high degree of cellular heterogeneity within the tissue. Only single cell analytics can resolve the cellular heterogeneity present in prostate tissue (Patel et al. 2014).

Repetitive single core biopsies of presumably the same location suffer from lack of accuracy due to uncontrollable distance between biopsy cores and cellular heterogeneity with high local variance. It also means repetitive trauma to the patient.

In patients not undergoing surgical therapy due to systemic disease such as metastatic prostate cancer, a fresh frozen biopsy core not only from the primary tumor but also from metastases provides an invaluable research source to further develop individualized therapy strategies. Little is known about microenvironment alterations under systemic therapy because tissue is rarely accessible with minimal risk to the patient. Additional components such as circulating tumor cells, exosomes or circulating RNA could complement the approach (Lohr et al. 2014; Peinado et al. 2012; Jackson et al. 2014). Advances in single cell technologies made this source accessible and will be the basis to develop novel diagnostic, prognostic and therapeutic tools.

## References

[CR1] Brennen WN, Isaacs JT, Denmeade SR (2012). Rationale behind targeting fibroblast activation protein-expressing carcinoma-associated fibroblasts as a novel chemotherapeutic strategy. Mol Cancer Ther.

[CR2] Comito G, Giannoni E, Segura CP, Barcellos-de-Souza P, Raspollini MR, Baroni G, Lanciotti M, Serni S, Chiarugi P (2014). Cancer-associated fibroblasts and M2-polarized macrophages synergize during prostate carcinoma progression. Oncogene.

[CR3] D’Amico AV, Whittington R, Malkowicz SB, Schultz D, Blank K, Broderick GA, Tomaszewski JE, Renshaw AA, Kaplan I, Beard CJ, Wein A (1998). Biochemical outcome after radical prostatectomy, external beam radiation therapy, or interstitial radiation therapy for clinically localized prostate cancer. JAMA.

[CR4] Donkor MK, Sarkar A, Savage PA, Franklin RA, Johnson LK, Jungbluth AA, Allison JP, Li MO (2011). T cell surveillance of oncogene-induced prostate cancer is impeded by T cell-derived TGF-beta1 cytokine. Immunity.

[CR5] Haffner MC, Mosbruger T, Esopi DM, Fedor H, Heaphy CM, Walker DA, Adejola N, Gurel M, Hicks J, Meeker AK, Halushka MK, Simons JW, Isaacs WB, De Marzo AM, Nelson WG, Yegnasubramanian S (2013). Tracking the clonal origin of lethal prostate cancer. J Clin Investig.

[CR6] Hedegaard J, Thorsen K, Lund MK, Hein AM, Hamilton-Dutoit SJ, Vang S, Nordentoft I, Birkenkamp-Demtroder K, Kruhoffer M, Hager H, Knudsen B, Andersen CL, Sorensen KD, Pedersen JS, Orntoft TF, Dyrskjot L (2014). Next-generation sequencing of RNA and DNA isolated from paired fresh-frozen and formalin-fixed paraffin-embedded samples of human cancer and normal tissue. PLoS One.

[CR7] Heidenreich A, Bastian PJ, Bellmunt J, Bolla M, Joniau S, van der Kwast T, Mason M, Matveev V, Wiegel T, Zattoni F, Mottet N, European Association of U (2014). EAU guidelines on prostate cancer. Part II: treatment of advanced, relapsing, and castration-resistant prostate cancer. Eur Urol.

[CR8] Heidenreich A, Bastian PJ, Bellmunt J, Bolla M, Joniau S, van der Kwast T, Mason M, Matveev V, Wiegel T, Zattoni F, Mottet N, European Association of U (2014). EAU guidelines on prostate cancer. Part 1: screening, diagnosis, and local treatment with curative intent-update 2013. Eur Urol.

[CR9] Jackson BL, Grabowska A, Ratan HL (2014). MicroRNA in prostate cancer: functional importance and potential as circulating biomarkers. BMC Cancer.

[CR10] Jiang Z, Woda BA, Yang XJ (2002). Alpha-methylacyl coenzyme A racemase as a marker for prostate cancer. Jama.

[CR11] Karja V, Aaltomaa S, Lipponen P, Isotalo T, Talja M, Mokka R (2005). Tumour-infiltrating lymphocytes: a prognostic factor of PSA-free survival in patients with local prostate carcinoma treated by radical prostatectomy. Anticancer Res.

[CR12] Kronig M, Walter M, Drendel V, Werner M, Jilg CA, Richter AS, Backofen R, McGarry D, Follo M, Schultze-Seemann W, Schule R (2015). Cell type specific gene expression analysis of prostate needle biopsies resolves tumor tissue heterogeneity. Oncotarget.

[CR13] Lohr JG, Adalsteinsson VA, Cibulskis K, Choudhury AD, Rosenberg M, Cruz-Gordillo P, Francis JM, Zhang CZ, Shalek AK, Satija R, Trombetta JJ, Lu D, Tallapragada N, Tahirova N, Kim S, Blumenstiel B, Sougnez C, Lowe A, Wong B, Auclair D, Van Allen EM, Nakabayashi M, Lis RT, Lee GS, Li T, Chabot MS, Ly A, Taplin ME, Clancy TE, Loda M, Regev A, Meyerson M, Hahn WC, Kantoff PW, Golub TR, Getz G, Boehm JS, Love JC (2014). Whole-exome sequencing of circulating tumor cells provides a window into metastatic prostate cancer. Nat Biotechnol.

[CR14] Madar S, Goldstein I, Rotter V (2013). ‘Cancer associated fibroblasts’–more than meets the eye. Trends Mol Med.

[CR15] Maher J, Davies ET (2004). Targeting cytotoxic T lymphocytes for cancer immunotherapy. Br J Cancer.

[CR16] Patel AP, Tirosh I, Trombetta JJ, Shalek AK, Gillespie SM, Wakimoto H, Cahill DP, Nahed BV, Curry WT, Martuza RL, Louis DN, Rozenblatt-Rosen O, Suva ML, Regev A, Bernstein BE (2014). Single-cell RNA-seq highlights intratumoral heterogeneity in primary glioblastoma. Science.

[CR17] Peinado H, Aleckovic M, Lavotshkin S, Matei I, Costa-Silva B, Moreno-Bueno G, Hergueta-Redondo M, Williams C, Garcia-Santos G, Ghajar C, Nitadori-Hoshino A, Hoffman C, Badal K, Garcia BA, Callahan MK, Yuan J, Martins VR, Skog J, Kaplan RN, Brady MS, Wolchok JD, Chapman PB, Kang Y, Bromberg J, Lyden D (2012). Melanoma exosomes educate bone marrow progenitor cells toward a pro-metastatic phenotype through MET. Nat Med.

[CR18] Pettit JB, Tomer R, Achim K, Richardson S, Azizi L, Marioni J (2014). Identifying cell types from spatially referenced single-cell expression datasets. PLoS Comput Biol.

[CR19] Picelli S, Bjorklund AK, Faridani OR, Sagasser S, Winberg G, Sandberg R (2013). Smart-seq2 for sensitive full-length transcriptome profiling in single cells. Nat Methods.

[CR20] Ramskold D, Luo S, Wang YC, Li R, Deng Q, Faridani OR, Daniels GA, Khrebtukova I, Loring JF, Laurent LC, Schroth GP, Sandberg R (2012). Full-length mRNA-Seq from single-cell levels of RNA and individual circulating tumor cells. Nat Biotechnol.

[CR21] Rubin MA, Zhou M, Dhanasekaran SM, Varambally S, Barrette TR, Sanda MG, Pienta KJ, Ghosh D, Chinnaiyan AM (2002). Alpha-Methylacyl coenzyme A racemase as a tissue biomarker for prostate cancer. JAMA.

[CR22] Schroder FH, Hugosson J, Roobol MJ, Tammela TL, Ciatto S, Nelen V, Kwiatkowski M, Lujan M, Lilja H, Zappa M, Denis LJ, Recker F, Paez A, Maattanen L, Bangma CH, Aus G, Carlsson S, Villers A, Rebillard X, van der Kwast T, Kujala PM, Blijenberg BG, Stenman UH, Huber A, Taari K, Hakama M, Moss SM, de Koning HJ, Auvinen A, Investigators E (2012). Prostate-cancer mortality at 11 years of follow-up. N Engl J Med.

[CR23] Sfanos KS, Bruno TC, Maris CH, Xu L, Thoburn CJ, DeMarzo AM, Meeker AK, Isaacs WB, Drake CG (2008). Phenotypic analysis of prostate-infiltrating lymphocytes reveals TH17 and Treg skewing. Clin Cancer Res.

[CR24] Shalek AK, Satija R, Shuga J, Trombetta JJ, Gennert D, Lu D, Chen P, Gertner RS, Gaublomme JT, Yosef N, Schwartz S, Fowler B, Weaver S, Wang J, Wang X, Ding R, Raychowdhury R, Friedman N, Hacohen N, Park H, May AP, Regev A (2014). Single-cell RNA-seq reveals dynamic paracrine control of cellular variation. Nature.

[CR25] Sluka P, Davis ID (2013). Cell mates: paracrine and stromal targets for prostate cancer therapy. Nat Rev Urol.

[CR26] Treutlein B, Brownfield DG, Wu AR, Neff NF, Mantalas GL, Espinoza FH, Desai TJ, Krasnow MA, Quake SR (2014). Reconstructing lineage hierarchies of the distal lung epithelium using single-cell RNA-seq. Nature.

[CR27] Trombetta JJ, Gennert D, Lu D, Satija R, Shalek AK, Regev A (2014) Preparation of single-cell RNA-Seq libraries for next generation sequencing. In: Ausubel FM et al (ed) Current protocols in molecular biology, vol 107, pp 4 22 21–24 22 17. doi:10.1002/0471142727.mb0422s107 10.1002/0471142727.mb0422s107 PMC4338574 24984854

[CR28] Webber JP, Spary LK, Sanders AJ, Chowdhury R, Jiang WG, Steadman R, Wymant J, Jones AT, Kynaston H, Mason MD, Tabi Z, Clayton A (2015). Differentiation of tumour-promoting stromal myofibroblasts by cancer exosomes. Oncogene.

[CR29] Zhou M, Chinnaiyan AM, Kleer CG, Lucas PC, Rubin MA (2002). Alpha-Methylacyl-CoA racemase: a novel tumor marker over-expressed in several human cancers and their precursor lesions. Am J Surg Pathol.

